# Effect of climatic variability on malaria trends in Baringo County, Kenya

**DOI:** 10.1186/s12936-017-1848-2

**Published:** 2017-05-25

**Authors:** Edwin K. Kipruto, Alfred O. Ochieng, Douglas N. Anyona, Macrae Mbalanya, Edna N. Mutua, Daniel Onguru, Isaac K. Nyamongo, Benson B. A. Estambale

**Affiliations:** 1 Hasselt University, Martelarenlaan 42, 3500 Hasselt, Belgium; 2 Division of Research Innovation and Outreach, Jaramogi Oginga Odinga University of Science and Technology, P. O. Box 210, Bondo, 40601 Kenya; 3 Institute of Anthropology, Gender and African Studies, University of Nairobi, P.O. Box 30197, Nairobi, 00100 Kenya; 4 School of Biological and Physical Sciences, Jaramogi Oginga Odinga University of Science and Technology, P. O. Box 210, Bondo, 40601 Kenya; 5 School of Health Sciences, Jaramogi Oginga Odinga University of Science and Technology, P. O. Box 210, Bondo, 40601 Kenya

**Keywords:** Baringo County, Malaria transmission, Seasonal trends, Rainfall, Temperature, Kenya

## Abstract

**Background:**

Malaria transmission in arid and semi-arid regions of Kenya such as Baringo County, is seasonal and often influenced by climatic factors. Unravelling the relationship between climate variables and malaria transmission dynamics is therefore instrumental in developing effective malaria control strategies. The main aim of this study was to describe the effects of variability of rainfall, maximum temperature and vegetation indices on seasonal trends of malaria in selected health facilities within Baringo County, Kenya.

**Methods:**

Climate variables sourced from the International Research Institute (IRI)/Lamont-Doherty Earth Observatory (LDEO) climate database and malaria cases reported in 10 health facilities spread across four ecological zones (riverine, lowland, mid-altitude and highland) between 2004 and 2014 were subjected to a time series analysis. A negative binomial regression model with lagged climate variables was used to model long-term monthly malaria cases. The seasonal Mann–Kendall trend test was then used to detect overall monotonic trends in malaria cases.

**Results:**

Malaria cases increased significantly in the highland and midland zones over the study period. Changes in malaria prevalence corresponded to variations in rainfall and maximum temperature. Rainfall at a time lag of 2 months resulted in an increase in malaria transmission across the four zones while an increase in temperature at time lags of 0 and 1 month resulted in an increase in malaria cases in the riverine and highland zones, respectively.

**Conclusion:**

Given the existence of a time lag between climatic variables more so rainfall and peak malaria transmission, appropriate control measures can be initiated at the onset of short and after long rains seasons.

## Background

Malaria is a global health problem that causes an estimated 438,000 deaths annually; 88% of which occur in the sub-Saharan Africa [1]. Seventy-five percent of the malaria clinical episodes worldwide occur in Africa with a corresponding high public health burden [2]. Up to 35.4 million disability adjusted life years (DALYs) are lost in the sub-Saharan Africa region alone due to malaria mortality and morbidity [3].

In Kenya, malaria is among the leading causes of morbidity and mortality and is responsible for almost half of all outpatient attendance and 20% of all admissions to health facilities [4]. Pregnant women and children under five years old are most vulnerable to malaria infections [5] with an estimated 170 million working days being lost to malaria in Kenya each year [6].

The high burden of malaria in Kenya and the larger sub-Saharan Africa region may be associated with a number of factors among them climatic and environmental [7]. Given that malaria is vector-transmitted, with a complex life cycle in both the mosquito and human, transmission and patterns of malaria infection are dependent on both environmental and climatic factors [8]. A study by Githeko et al. [9] showed that inter-annual and inter-decadal climate variability influences the epidemiology of vector-borne diseases directly, while temperature and rainfall have long been known to influence seasonal and inter-annual variability of malaria [10].

The effect of temperature on the life history traits of mosquitoes and malaria transmission has been reported. Temperature can affect the development time of mosquito larvae, the probability of mosquito survival and the development time of malaria parasite *(Plasmodium falciparum*) in infected mosquitoes either positively or negatively [11]. A rise in temperature to a certain threshold can accelerate the metabolic rate of vectors, increase egg production and increase frequency of blood meals, while temperatures below or above these thresholds can be detrimental to mosquitoes and parasite development [12]. Several mechanistic models concur that effect of temperature on malaria transmission is non-linear, limited to temperatures between 16 and 34 °C with a peak at 25 °C [13–15]. The non-linear temperature sensitivities throughout the mosquito life cycle have a large impact on the adult population dynamics and, therefore, on the mosquitoes’ ability to act effectively as malaria vectors.

Rainfall influences vector longevity indirectly by creating wet conditions that favour vector breeding. This in turn influences the geographical range and seasonal variability of disease vectors [16]. The relationship between malaria incidence and rainfall is non-linear, implying that an increase in precipitation would not necessarily increase malaria cases [17]. Moderate rainfall has a positive effect on mosquito abundance, while intense precipitation can wash away mosquito breeding sites, and therefore reduce malaria transmission shortly following heavy rains [18]. The Normalized Difference Vegetation Index (NDVI) is a spectral measure of amount, relative greenness, phenological characteristics and productivity of vegetation [19]. It is defined as the difference between the visible (RED) and near-infrared (NIR) bands over their sum, (*NIR−RED*)/(*NIR* + *RED*). It is a robust indicator of vegetation condition which allows valid comparisons of seasonal and inter-annual variations in vegetation growth and activity [20]. In the study area, the seasonal NDVI variations are linked to rainfall. NDVI values range between −1 to +1 An NDVI value of zero means no green vegetation and close to +1 (0.8–0.9) indicates the highest possible density of green leaves. NDVI can be used as a surrogate for precipitation based on their close correlation [21]. The capability of NDVI time-series to monitor and predict vector-borne diseases depends on the correlation between disease incidence, vegetation greenness and precipitation [22].

The nature of vector biological processes and the degree to which the vectors depend on environmental and climatic factors makes malaria transmission somewhat region specific [23]. In Kenya, there are four epidemiological zones whose diversity in malaria transmission and risk is determined by altitude, rainfall patterns and temperature. The zones include: the endemic Lake Victoria and coastal regions, epidemic-prone Western highlands, seasonal transmission arid and semi-arid areas, and low risk central highlands [24]. Parts of Baringo County being semi-arid experience seasonal malaria transmission [25], while the presence of numerous seasonal and permanent water bodies provides suitable breeding microhabitats for malaria vectors at certain times of the year.

This study was thus necessitated by the lack of information on the interactions of climatic and environmental factors, and their role in driving the transmission and prevalence of malaria across the different ecological zones of Baringo County, which has been hampering the planning of intervention strategies against malaria. The study modelled the effect of climatic variations on the prevalence and long-term trend of malaria so as to identify the seasonal climatic drivers of malaria transmission in the different ecological zones of Baringo County.

## Methods

### Study area

**Fig. 1:**
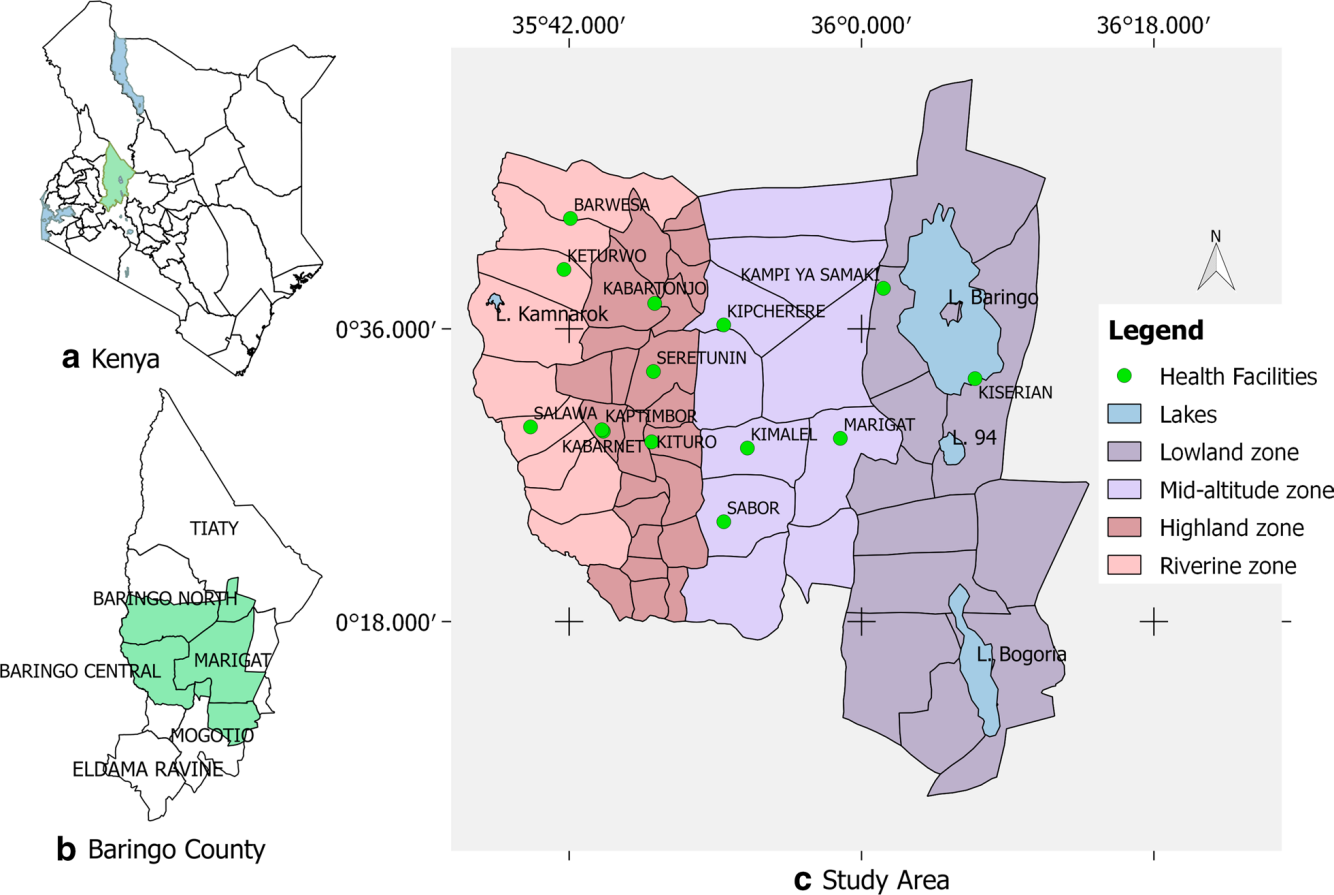
**a** Map of the study area showing the location of Baringo County in Kenya, **b** the sub-county administrative units within Baringo County with the study area shaded out *green*, and **c** the ecological zones within the study area and the health facilities from which malaria prevalence data was collected

This study focused on Baringo County, Kenya, located between 35.602°E, 36.277°E and 0.541°N, 0.723°N, at altitudes ranging between 870 and 2499 m above sea level (asl). The county covers 11,075 km^2^ with an estimated population of 555,561 as at 2009 [26]. The average annual rainfall ranges between 300 and 1500 mm while air temperature varies between 16 °C in the highland areas and 42 °C in the lake ecosystem [27]. Rainfall pattern is tri-modal, with the long rains received during March–May (MAM) and two short rains seasons that are experienced between June–August (JJA) and October–December (OND). This study focused on the central part of Baringo County (Fig. 1) encompassing three sub-counties (Baringo North, Baringo Central and Baringo South). The study area was sub-divided into four ecological zones on the basis of hydrology, altitude, vegetation cover, soil types, and precipitation. The four zones were the lowland zone lying at an elevation of 1000 m asl and surrounding the permanent water bodies (L. Baringo, L. Bogoria and L. 94), the mid-altitude zone lying between 1000 and 1500 m asl, the highland zone, lying between 1,500 and 2,300 m asl and the riverine zone (bordering the Kerio River) at altitudes ranging between 1100 and 1200 m asl [28].

### Selection of health facilities

Ten health facilities were selected across the four zones based on availability of health records and the catchment population served; with those serving larger populations being selected. The facilities included: Kabarnet County Hospital, Kituro Health Centre and Kabartonjo sub-county Hospital in the highlands; Sabor Dispensary and Kipcherere Health Centre in the mid-altitudes; Marigat sub-county Hospital and Kampi ya Samaki Health Centre in the lowlands; and Barwessa, Salawa and Keturwo Health Centres in the riverine zone. The available datasets varied per region. The highland and mid-altitude zones had data spanning for a period of 10 years (2004–2013) while lowland (2005–2013) and riverine (2006–2014) zone had data spanning 9 years.

### Retrospective data extraction

#### Malaria data

Retrospective health records of clinically-diagnosed and treated malaria cases between 2004 and 2014 were extracted from the registers in the 10 health facilities. Daily counts of malaria cases among all age groups and gender were entered in MS Excel and computed into monthly data sets for analyses. Only two out of the 10 health facilities that were studied had some missing data, i.e. Marigat sub-county Hospital which had 13% (14/108 months) of the data missing and Keturwo Health Centre which had 8.3% (9/108 months) of the data missing. The missing data were imputed on aggregated monthly data sets using predictive mean matching method using mice package in R 3.0.3 statistical software [29].

#### Imputation of missing data

The unavailable monthly data was assumed to be missing at random (MAR) before multiple imputation was conducted. Predictive mean matching (PMM) method was used to enable imputation of the missing values based on the observed values. Through this method the missing values were imputed by means of nearest neighbour values with distances based on the expected values of the missing variables [30]. Unlike other imputation methods, the PPM method produces acceptable estimates and preserves the underlying distribution of the observed data especially for quantitative variables that are not normally distributed. Compared with other methods, PMM produces imputed values that are much more like real values. The major pitfall with PMM however is that there is no mathematical theory to justify it and only a handful studies have evaluated its performance, meaning that it is still not clear how well it compares with alternative methods [31].

#### Climate and environmental data

Monthly average rainfall, inferred maximum air temperatures and enhanced vegetation indices over the study period were sourced from the International Research Institutes of Climate and Society’s database [32]. The monthly average rainfall data used was obtained from University of California Santa Barbara (UCSB) Climate Hazards Group InfraRed Precipitation with Station Data (CHIRPS) v2p0 [33]. Annual precipitation was averaged from monthly estimates for each year. Inferred maximum air temperature (Tmax) and minimum land surface temperature (Tmin) used as proxy for minimum and maximum air temperatures were obtained from the United States Geological Survey (USGS) LandDAAC MODIS 1 km 8 day version 005 datasets [34, 35]. Sixteen-day MODIS Enhanced Vegetation Index (EVI) composites from MODIS-Terra MOD13Q1 at 250 m spatial resolution [35] were used to derive 15 years temporal profiles.

For all data sets, the spatial averages limited to the spatial extent of the ecological zones were downloaded in expert mode. The 8- and 16-day composites were averaged into monthly means for each year.

#### Data analysis

Exploratory data analysis was used to visualize seasonal patterns of climate variables in relation to malaria cases across the four zones. A smoothing line was added to the patterns to get good visual information. Locally weighted regression was used to smoothen the data points with a smoother span of 0.67, tricube as weighted function, number of iterations for robust fitting 3 and an order of the polynomial of 1.

The Seasonal Mann–Kendall trend test was used to detect malaria monthly trends over the study period. Pair wise comparison of monthly means of malaria cases was carried out in order to draw simultaneous inference about the dominant malaria seasons using Tukey multiple comparison procedure. Additive decomposition of malaria cases and climatic variables were conducted in order to estimate the trend component. The trend was determined using moving averages as the smoothing method. The sample cross correlation function was used to identify lags of climate variables that were useful predictors of malaria cases. Dominant cross correlations between malaria cases and climate variables were selected and included in the regression model. A negative binomial regression model with lagged climate variables was used to model the monthly malaria cases. Model fit was assessed by checking the autocorrelation function and partial autocorrelation function of the model residuals. Variance inflation factors (VIF) were computed to check multi-collinearity. The analyses were done using dyn and trend packages in R [36, 37].

#### Ethics statement

Ethical approval to access hospital registers for data extraction was obtained from the Kenyatta National Hospital/University of Nairobi Ethical Review Committee (P70/02/2013) and also from the Ministry of Health (Ref.: CNTY/GEN/Vol.1/83) and Department of Medical Services (Ref: BCG/CDH/GEN/VOL.II/2015), Baringo County.

## Results

### Long-term mean monthly malaria cases (2004–2014)

The long-term (2004–2014) mean monthly malaria cases varied across zones. On average, the observed monthly malaria cases were highest in the highlands and lowest in mid-altitudes. Low variability was observed in midland zone while high variability was observed in the other three zones. Highest and lowest malaria cases per zone were however recorded in different months and years over the study period. Table 1 summarizes the data for each zone over the study period.

**Table 1:** Long-term malaria cases (2004–2014)

Zones	Overall mean	SE	Highest cases (month, year)	Lowest cases (month, year)
Highland	694.25	19.8	1799 (Oct 2012)	285 (May 2009)
Mid-altitude	65.9	2.8	197 (Oct 2009)	20 (Apr 2006)
Lowland	577.5	22.4	1318 (Aug 2006)	126 (Jan 2005)
Riverine	434	16.3	979 (Nov 2009)	110 (May 2014)

The means and SE relate to monthly malaria cases over the 2004–2014 period

### Yearly cumulative malaria cases (2004–2014) and trends

**Fig. 2:**
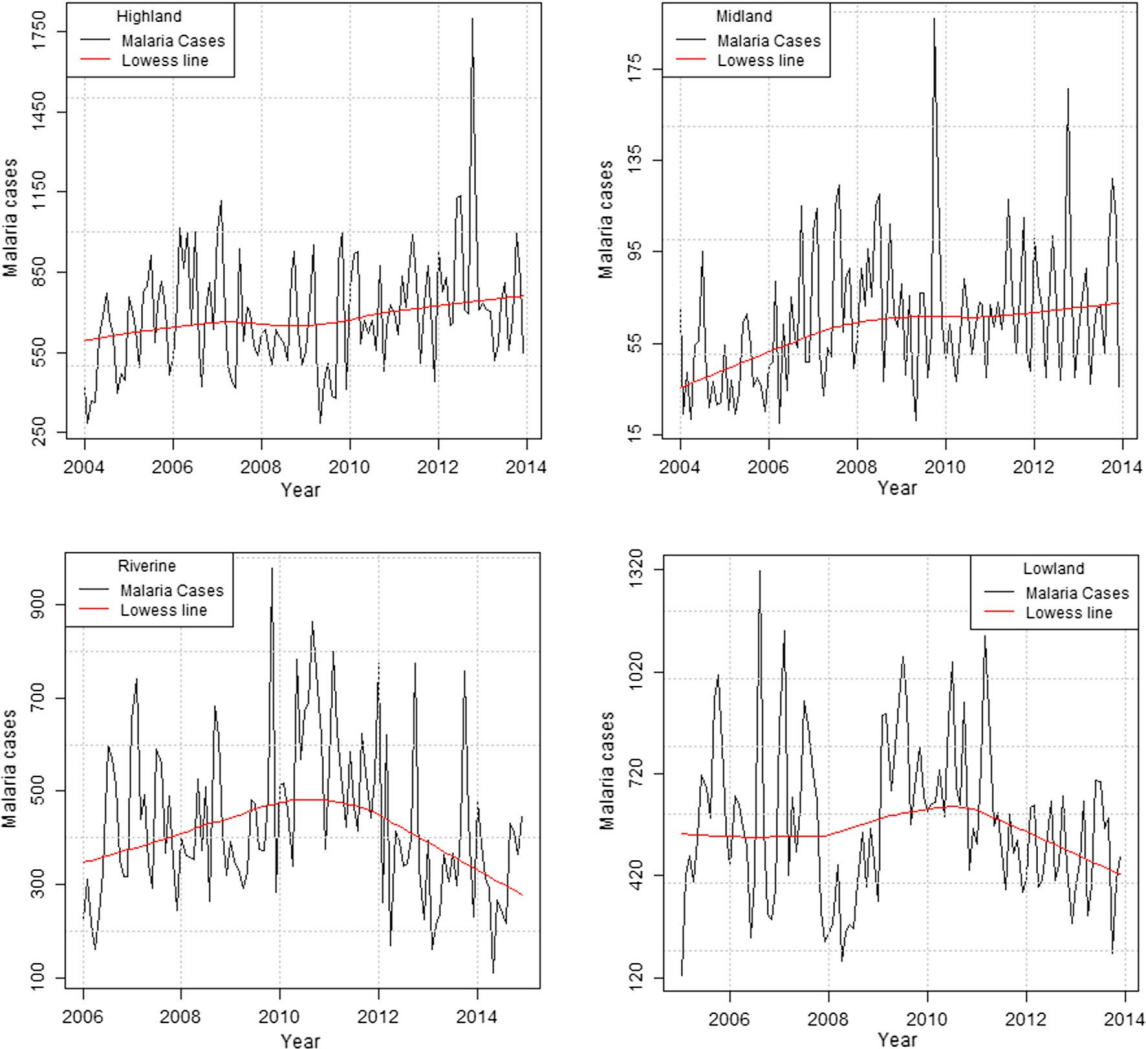
Long-term yearly malaria cases pattern with a *lowess smoothline* in the highland, mid-altitude, lowland and riverine zones

The highest yearly cumulative malaria cases were recorded in 2012 (11,249 cases) in the highland, 2008 (971 cases) in the mid-altitude, 2009 (9275 cases—over 9 year period) in the lowland and 2010 (7119 cases—over 9 year period) in the riverine zone. Health records for the year 2004 in the lowland and the year 2004 and 2005 in the riverine zone were however not available. The Seasonal Mann–Kendall trend test showed a significant increasing trend in malaria cases over the study period in the highland (z = 2.5, p = 0.0142) and mid-altitude (z = 2.5, p = 0.0141) zones while no change was observed in the lowland (z = −0.5, p = 0.609) and riverine zones (z = −0.6, p = 0.567) (Fig. 2). However, further analysis showed a significant decrease in malaria cases in the riverine zone between 2011 and 2014 (z = −3.2, p = 0.0012).

### Malaria seasonality over 10 year period

**Fig. 3:**
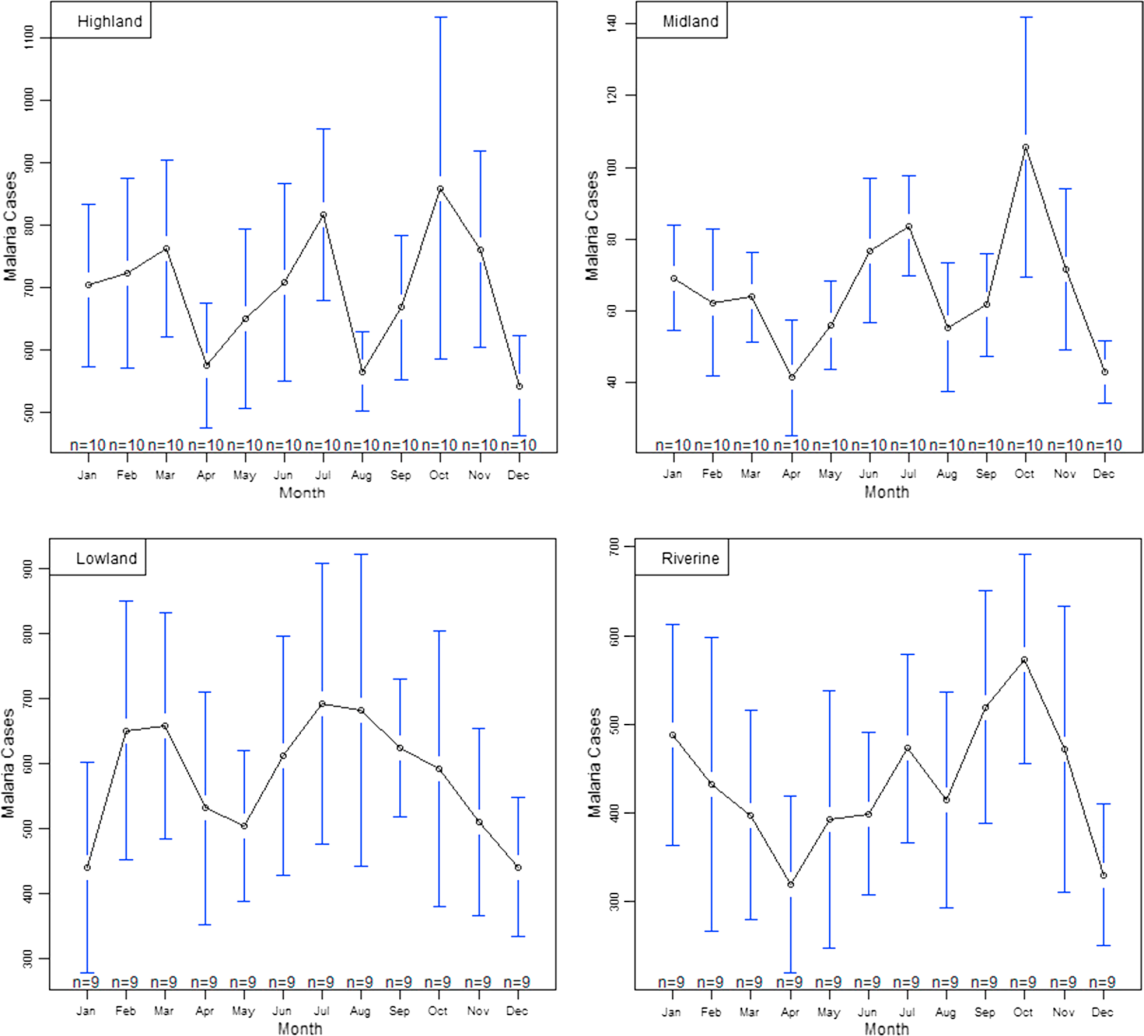
Monthly averages with 95% confidence intervals depicting malaria peak seasons over the study period (2004–2014)

Three malaria peak seasons were observed in the riverine, highland and mid-altitude zones, while two seasons were observed in the lowland zone (Fig. 3). Pair wise mean comparison tests for the monthly malaria cases in each zone yielded different results. In the highland zone, the month of October experienced significantly higher malaria cases compared to December (estimate = 315.8, CI 9.95–621.65). Mean differences for the other months were statistically insignificant. In the mid-altitude zone, there were significant differences in mean monthly malaria cases for the month of July and April (estimate = 42.3, CI 3.06–81.54), July and December (estimate = 40.7, CI 1.46–79.94), October and February (estimate = 43.4, CI 4.16–82.64), October and March (estimate = 41.7, CI 2.46–80.94) and, October and August (estimate = 50.2, CI 10.96–89.44). The months of July and October recorded significantly higher malaria cases in the highland and mid-altitude zones, a period corresponding to the short rainy seasons of June–August and October–November. There were however no significant differences in mean monthly malaria cases in the lowland and riverine zones.

## Relating malaria cases to climatic variables

### Long-term trends of malaria cases against climatic variables

**Fig. 4:**
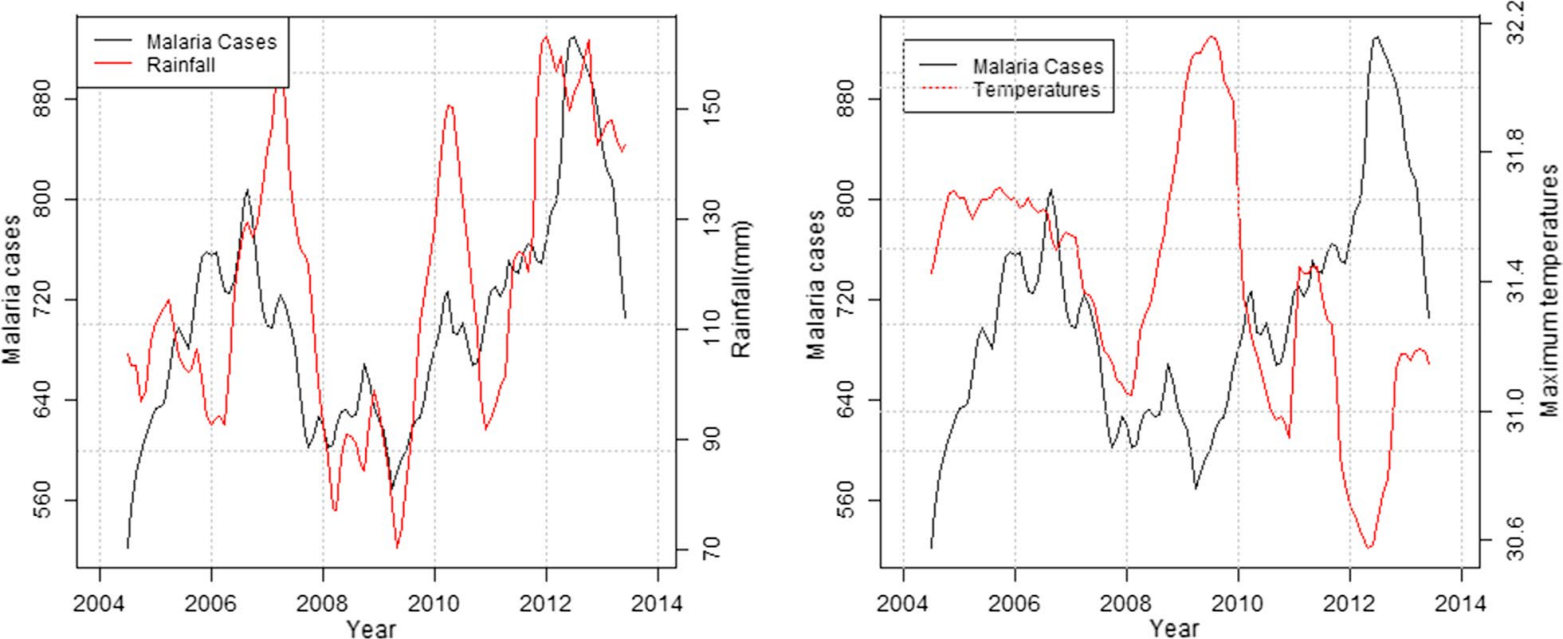
Long-term trends in malaria cases against rainfall and maximum temperature in the highland zone (2004–2013)

**Fig. 5:**
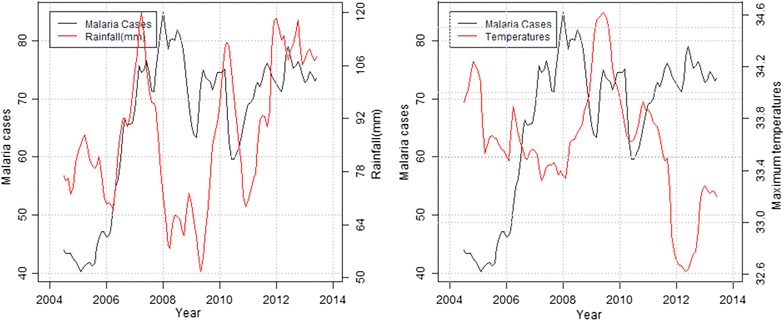
Long-term trend of malaria cases against rainfall and temperatures in the mid-altitude zone (2004–2013)

**Fig. 6:**
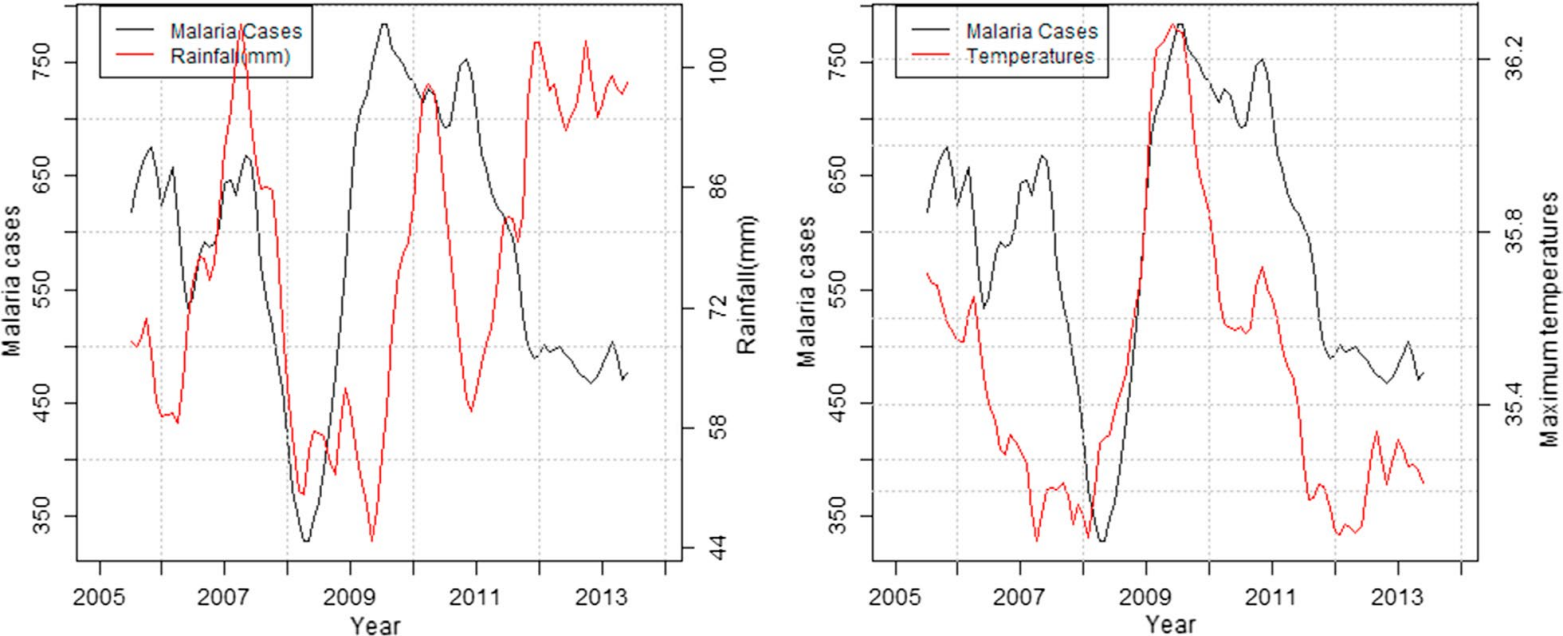
Long-term trend of malaria cases against rainfall and temperatures in the lowland zone (2005–2013)

**Fig. 7:**
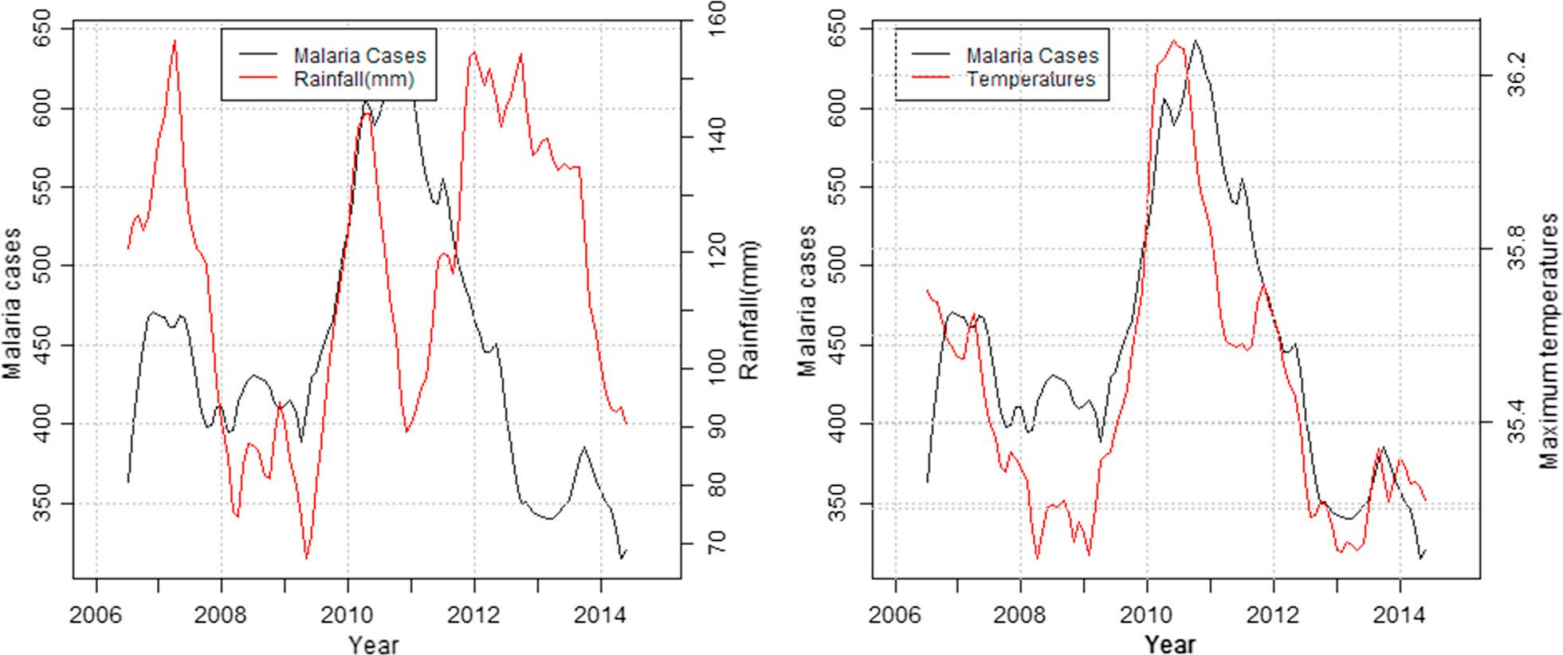
Long-term trend of malaria cases against rainfall and temperatures in the riverine zone (2006–2014)

In the highland zone, peaks in malaria cases followed low peaks of rainfall while declines were associated with high peaks of rainfall and decrease in maximum temperatures. Decline in malaria cases between September and December 2006 corresponded to heavy precipitation and reduced maximum temperature recorded the previous months (Fig. 4). In the mid-altitude zone, malaria cases and rainfall followed a similar upward trend between 2006 and 2007, before declining between 2008 and 2009 following extremely low rainfall amounts and increased maximum temperatures recorded over the same period. Again in 2012, malaria cases rose following increased rainfall and slight increase in maximum temperatures recorded that year (Fig. 5). In the lowland zone, malaria cases increased following an increase in rainfall and a decrease in maximum temperature between 2006 and 2007. A sharp decline in malaria cases was however noted between 2007 and 2008 following high rainfall and low temperatures (Fig. 6). In the riverine zone, malaria cases increased between 2006 and 2007 and again between 2009 and 2010 following increased rainfall, while a decrease in malaria cases was reported between 2011 and 2013, corresponding to high rainfall amounts and low temperatures received over the same period (Fig. 7).

### Identification of possible climatic and environmental predictors of malaria cases

Using the sample cross correlation function, it was established that rainfall at lags 1 and 2 months across all zones and also at lag 0 in the lowland zone had the most dominant cross correlation with malaria cases. Temperature at lag 0 in the lowland and riverine zones, and lag 1 in the highland, mid-altitude and riverine zones had the most dominant cross correlation with malaria cases. Enhanced Vegetation Index at lag 0 in the highland, mid-altitude and lowland zones and lag 1 and 2 in the highland and riverine zones had the most dominant cross correlations with malaria cases. Based on the CCF, a negative binomial regression model was built, and it was observed that rainfall lagging 2 months in all the zones were statistically significant at 5% level of significant. However, rainfall in midland was highly significant as compared to the other zones. Further it was observed that maximum temperature at lag 0 in the riverine zone and lag 1 in the highland zone were significant. Joint significance of rainfall and temperatures in highland and riverine can be attributed to high variations in malaria cases in these zones (Table 2).

**Table 2:** Rainfall, temperature and EVI lags in relation to malaria cases in the four zones

	Estimate	Std. error	Z value	p value
Highland				
Rainfall (lag 1)	−0.0004	0.00047	−0.871	0.3838
Rainfall (lag 2)	0.0009	0.00045	2.078	0.0378*
Temperature (lag 1)	0.0387	0.01700	2.278	0.0227*
EVI (lag 0)	0.6225	1.08295	0.575	0.5654
EVI (lag 1)	1.6205	1.27888	1.267	0.2051
EVI (lag 2)	−1.3151	0.91188	−1.442	0.1493
MID-altitude				
Rainfall (lag 1)	−0.0012	0.00121	−0.964	0.3352
Rainfall (lag 2)	0.0025	0.00080	3.140	0.0017**
Temperature (lag 1)	0.0074	0.02860	0.259	0.7954
EVI (lag 0)	0.3339	1.14059	0.293	0.7697
Lowland				
Rainfall (lag 0)	0.0013	0.00100	1.299	0.1938
Rainfall (lag 1)	0.0010	0.00127	0.810	0.4179
Rainfall (lag 2)	0.0018	0.00089	2.026	0.0427*
Temperature (lag 0)	0.0547	0.03213	1.703	0.0886
EVI (lag 0)	−1.5132	1.43254	−1.056	0.2908
Riverine				
Rainfall (lag 1)	0.0005	0.00070	0.746	0.4553
Rainfall (lag 2)	0.0016	0.00080	1.966	0.0493*
Temperature (lag 0)	0.0631	0.02262	2.788	0.0053**
Temperature (lag 1)	−0.0120	0.02784	−0.431	0.6662
EVI (lag 1)	−0.7258	1.77546	−0.409	0.6827
EVI (lag 2)	0.6631	1.14723	0.578	0.5633

Significance codes: * p value ≤0.05, ** p value ≤0.01

## Discussion

Climatic factors are considered important in the spatial and temporal distribution of vector borne diseases as they determine vector distribution, and influence inter-annual variability, epidemics and long-term trends [15]. There is a strong discernible link between malaria outbreaks, temperature [13] and rainfall [38]. In the current study, malaria cases generally increased in highland and mid-altitude zones but decreased in the riverine and lowland zones from the year 2011 onwards during the study period (Fig. 2). Two malaria peak seasons were identified in the lowland zone while three malaria peak seasons were identified in the other zones, largely following climatic seasons in the study area. Statistically significant differences in monthly malaria peaks was recorded in the highlands and mid-altitude zones suggesting seasonal malaria transmission. However there was no statistical significance in malaria peaks in the lowland and riverine zones, suggesting that malaria transmission in these two zones is perennial rather than seasonal as previously thought [24].

The mean monthly rainfall for this study showed positive significant correlation with malaria cases at 2 months lag across all zones while the mean maximum temperature showed positive significant correlation with malaria cases in two zones, the highlands and the riverine zones. Previous studies examining the link between climate and malaria established lagged associations between climate variables (temperature and rainfall) and malaria cases over time periods ranging from weeks to months [10, 18, 39–42]. These studies attributed the lags to the creation of mosquito breeding habitats, the time required by mosquitoes to develop to adulthood, acquire and transmit malarial infection, and for symptoms to arise in the human host as the most probable cause.

According to Confalonieri et al. [43], periods of unusually high rainfall, altered humidity or warmer temperatures can result in modified distribution and duration of malaria, as well as increased transmission; even in areas where control is strong. Consistent with the current findings, Small et al. [44] cited precipitation and temperature as key drivers of malaria case variations across Africa, while acknowledging the complexities of some climatic factors.

The difference in environmental relationship to malaria cases across the zones is attributed to variations in environmental factors between the zones. The mid-altitude zone has no rivers or water bodies and rainfall is therefore the only source of surface water that serves as breeding points for malaria vectors. Although the other zones have permanent water bodies in the form of lakes, rivers, swamps, dams and water pans, rainfall still contributes to malaria cases through creation of additional seasonal breeding sites for malaria vectors.

Consistent with our study findings, Chaves et al. [38] cited increased microhabitats resulting from relative humidity caused by moderate rainfall. These conditions increase the longevity of adult mosquitoes by prolonging vector life span. Paaijmans et al. [45] highlighted the complex interrelationship between precipitation and vectors, noting that drought may eliminate mosquito habitats, while floods could create isolated pools suitable for vector breeding. The relatively low annual rainfall in the mid-altitude zone and the general absence of permanent water bodies contributed to the observed low but varying numbers of recorded malaria cases; possibly due to the varying climatic conditions. All in all, there is a general consensus that rainfall can influence malaria transmission either positively by creating suitable habitats or negatively by flushing breeding sites depending on its intensity [46, 47].

Temperature plays a key role in malaria transmission by influencing vector and parasite life cycles. Studies have highlighted the biological amplification nature of temperature on mosquitoes [48]. This study showed that the mean maximum temperatures within the four zones varied. While the mean maximum temperature significantly influenced malaria cases at lag 0 in the riverine zone and lag 1 in the highlands, it was non-significant in the mid-altitude and lowland zones. The difference in the contribution of maximum temperature to malaria cases between zones is attributed to the differences in prevailing temperatures in the four zones. Being colder, temperature was probably the limiting factor in malaria vector development in the highland and riverine zones; hence a rise in the maximum temperature increased vector and parasite development rates [40]. Since temperature influences the development and survival rates of both vectors and parasites, malaria transmission rates tend to increase with increasing temperature but up to a given threshold [49].

Craig et al. [14] put the optimal temperatures for malaria transmission at between 22 and 32 °C, while Bi et al. [50] reported temperatures of between 20 and 30 °C as being optimal for *Anopheles* survival and that temperatures below 16 °C and above 30 °C have a negative impact on mosquitoes survival. Chikodzi [51] noted that temperatures above 32 °C can cause high vector population turnover, with thermal death for mosquitoes expected to occur around 41–42 °C.

Vegetation index often acts as a surrogate for precipitation and surface temperatures and has been correlated to vector borne diseases [22]. In this study, vegetation cover followed a positive trend with the amount of precipitation received. In this study, EVI did not play any significant role in malaria transmission across the four zones.

## Conclusion

This study established seasonality in malaria transmission over the study period (2004–2014) in the highland and mid-altitude zone. Malaria transmission in the lowland and riverine zone was shown to be perennial. Peak malaria cases followed increased rainfall with a time lag of 2 months across the study area and increased maximum temperatures with a time lag of 0 and 1 months in the riverine and highland zones respectively. The observed time lags between peak malaria cases and climatic variables are particularly important in forecasting malaria outbreak using local weather data. Therefore, monitoring rainfall and temperature trends and early recognition of anomalies in weather patterns can provide a fairly accurate forecast of transmission risk within Baringo County, and hence inform timely action including vector control measures.
